# EFFECTIVENESS OF A DEMENTIA CARE INTELLIGENT RECOMMENDER SYSTEM ON CARE PROBLEMS: A RANDOMIZED CONTROLLED TRIAL

**DOI:** 10.1093/geroni/igae098.0733

**Published:** 2024-12-31

**Authors:** Yue Sun, Zhi-wen Wang

**Affiliations:** Peking University, Beijing, Beijing, China (People’s Republic); Peking University Health Science Center, Beijing, Beijing, China (People’s Republic)

## Abstract

**Background:**

The complexity and diversity of problems encountered in dementia care varies from the individual, and the health recommender system provides a pathway for realizing solutions for universal access to professional guidance.

**Objectives:**

To examine the effectiveness of dementia care intelligent recommender system (DCIRS) for dementia care on improving the number of care problems and their level of distress.

**Methods:**

250 caregivers of dementia patients were included and randomly assigned them to either the intervention group receiving the intervention of DCIRS or a waiting-list control group with 12 weeks of intervention length. The number of care problems and their level of distress was measured using the care problems evaluation sheet for people with dementia. The outcomes were compared between groups by using generalized estimating equations (GEE) based on intention-to-treat principle.

**Results:**

A total of 250 participants were included in the analysis. The dietary, dressing, and excretory care problems of the patients with dementia in the control group significantly increased at T2, while there was no significant change in the number of care problems in the intervention group. GEE results showed that participants in the intervention group revealed significantly greater reductions on the level of distress related to care problems than the control group at T1 (β = −3.01, p = 0.026) and T2 (β = −5.80, p = 0.006). Conclusions: The DCIRS was effective in improving the number of care problems and their level of distress. Longer follow-up study is needed to uncover its long-term benefits on clinical outcomes.

